# A higher TyG index is related with a higher prevalence of erectile dysfunction in males between the ages 20-70 in the United States, according to a cross-sectional research

**DOI:** 10.3389/fendo.2022.988257

**Published:** 2022-09-08

**Authors:** Lin Li, Hui Yao, Wei Dai, Yan Chen, Heqian Liu, Wei Ding, Yingqing Liu, Lingsong Tao, Jiawei Wang, Mingwei Chen

**Affiliations:** ^1^ Department of Urology, Wuhu City Second People’s Hospital, Wuhu City, China; ^2^ Department of Geriatrics, Wuhu City Second People’s Hospital, Wuhu City, China; ^3^ Department of General Practice, Wuhu City Second People’s Hospital, Wuhu City, China; ^4^ Department of Endocrinology, The First Affiliated Hospital of Anhui Medical University, Hefei City, China

**Keywords:** cross-sectional study, NHANES, erectile dysfunction (ED), insulin resistance, metabolic syndrome, triglyceride glucose index (TyG index)

## Abstract

**Objective:**

This study aims to investigate the relationship between triglyceride glucose index (TyG) and erectile dysfunction (ED) among United States (US) adult males.

**Methods:**

A logistic regression analysis, subgroup analysis, and the computation of the dose-response curve were used to investigate the relationship between TyG index and ED prevalence among participants from the 2001-2004 National Health and Nutrition Examination Survey (NHANES) database.

**Results:**

After adjusting for all confounders, each unit increase in TyR index was associated with a 25 percent increase in ED prevalence (OR=1.25, 95%CI:1.03, 1.52), and stratified analysis showed that elevated TyG index was associated with increased ED prevalence in the 50-year old group (OR=1.35, 95% CI:1.05, 1.74), the Mexican-American group (OR=1.50, 95% CI:1.00, 2.23) and BMI 25-29.9 kg/m^2^ (OR=1.48, 95% CI:1.08, 2.01). The dose-response curve demonstrated a positive linear connection between the TyG index and the risk of ED.

**Conclusion:**

It has been shown that a higher TyG index is associated with a higher prevalence of erectile dysfunction. Although the causal relationship is not clear, it still deserves clinical attention

## Introduction

Erectile dysfunction is defined by an international medical consultation as the chronic and recurring inability to obtain or sustain an erection that is sufficiently large and long to support sexual activity (1).As erectile dysfunction is a common disorder in older men, the Massachusetts Male Aging Study (MMAS) and the European Male Aging Study (EMAS) found that 52% of men between the ages of 40 and 70 have mild to moderate erectile dysfunction, and that erectile dysfunction is strongly correlated with age, health status, and emotional functioning (2). According to the European Multicenter Population Study (EMAS), which conducted the largest study of males with aging populations (40–79), erectile dysfunction prevalence ranged from 6% to 64%, depending on age grouping, and increased with age, averaging 30% (3). As of today, the most common treatment of erectile dysfunction involves lifestyle changes that are beneficial to the patient’s overall health and sexual function. Aside from behavior modification, other treatment options include transrectal thermotherapy (TRT), PDE5 inhibitors, intracavernosal injection therapy, vacuum constriction devices (VCD), intraurethral prostaglandin suppositories, and surgical placement of prosthetic devices (4). Although effective oral and injectable drugs have been introduced for the treatment of erectile dysfunction, these medications have not been demonstrated to restore natural erectile physiology, and in some cases these drugs are contraindicated, intolerable, expensive, or ineffective. The mechanical replacement of functional abilities by external and internal devices has proved beneficial for a number of men, yet it is often less satisfying than natural function and carries risks. It is important to note that despite the effectiveness of these interventions, the functional improvement in erection is sometimes limited and is often insufficient to satisfy the demands of the majority of patients (5). Identifying risk factors for erectile dysfunction, including as smoking, obesity, sedentary lifestyle, and persistent alcohol intake, is crucial for preventing erectile dysfunction (6–8).

Due to the prevalence of erectile dysfunction and its increasing occurrence among young men (9), prevention of erectile dysfunction, which may be directly related to metabolic psychospiritual culture, should be a priority (10). Modifiable risk factors for erectile dysfunction include central obesity, hypertension (HTN), dyslipidemia, and poor glucose tolerance, as well as metabolic syndrome, lack of exercise, and smoking (11). Moreover, correlational research indicates that erectile dysfunction is a correlate marker of cardiovascular disease and metabolic diseases in young men (12). According to studies, the prevalence of ED among patients with metabolic syndrome is three times higher (13–15). The metabolic syndrome is characterized by insulin resistance (IR), a central mechanism that results in endothelial dysfunction due to a reduction in NO synthesis and release, combined with a higher NO consumption in tissues which are exposed to high levels of reactive oxygen species (16). Vascular endothelial dysfunction plays a central role in the development of ED (17). IR has been demonstrated to induce vascular endothelin-b receptor in insulin-resistant obese mice, which is involved in the formation of increased reactive oxygen species, endothelial dysfunction, and enhanced vasoconstriction in erectile tissue (18). Through a negative feedback loop, this modification in endothelial function, particularly in arteries and capillaries, decreases insulin metabolic function.

Currently, the glucose clamp is the gold standard method for detecting IR. The Homeostatic Model Assessment (HOMA-IR) and the Quantitative Insulin Sensitivity Check method (QUIC) are alternatives to the glucose clamp method for determining IR using insulin and glucose levels (19, 20), but it is difficult for most laboratories to perform insulin testing in underdeveloped countries, limiting the indicators’ applicability. Several recent studies have suggested the triglyceride-glucose (TyG) index, which is computed using fasting triglyceride (TG) and glucose levels, as a simple, reliable, and repeatable index marker for measuring insulin resistance (IR) (21, 22). Since the TyG index has been presented as a marker for IR, one may speculate a link between the TyG index and ED. Currently, there is only one report on the relationship between TyG index and ED, Yilmaz (23) et al. reported a small sample study of 152 cases, concluding that TyG index may contribute to the diagnosis and follow-up of ED, then limited by the sample size, we expect to better elaborate the relationship between TyG index and ED by expanding the sample size and including more covariates.

## Materials and methods

### Study population

The data used in this study were obtained from the NHANES database. NHANES is conducted annually by the National Center for Health Statistics (NCHS), a division of the Centers for Disease Control and Prevention CDC, to assess the health nutritional status and health behaviors of unstructured populations in the US (24).A complex multistage probability sampling design was used in the NHANES survey to obtain representative data (25).

The NHANES survey employed a complicated multistage probability sampling methodology to acquire representative data (22). All NHANES procedures were implemented in compliance with the Human Research Subject Protection Policy of the U.S. Department of Health and Human Services (HHS) and were reviewed and standardized yearly by the NCHS Research Ethics Review Committee. All individuals who participated in the survey completed a permission form indicating their knowledge of the study’s purpose.

In this cross-sectional analysis, we chose datasets from two NHANES survey cycles (2001-2002 and 2003-2004). This is due to the fact that only these two cycles have ED and TyG index values. From 2001 to 2004, a total of 21161 individuals participated in NHANES;.Exclusion criteria were as follows: 1. females (n=10860); 2. missing ED information (n=6183); 3. aged>70 (n=701); 4. missing education level (n=2); 5. missing marital information (n=2); 6. missing TyG index information (n=`168); 7. missing hypertension information (n=51) 8. missing information about diabetes (n=1); 9. missing information about smoking (n=3); 10. missing information about asthma (n=7); 11. missing information about alcohol (n=2); 12. missing HDL-c information(n=3);13.missing coronary artery disease(n=12). Finally a total of 3166 cases were included in this study, including 606 self-reported ED history.

### Data collection and definition

The TyG index of triglycerides was created as an exposure variable.TyG index =Ln[fasting triglyceride (mg/dL)  × fasting glucose (mg/dL)/2],. Blood samples were processed to determine fasting glucose and fasting total triglyceride levels in the morning after 8.5 hours of fasting. Triglyceride and fasting blood glucose concentrations were determined enzymatically using an automated biochemical analyzer. Serum triglyceride concentrations were measured using the Roche Modular P and Roche Cobas 6000 chemistry analyzers. Self-report questionnaires and patients using sildenafil (only 4 participants) were used to diagnose erectile dysfunction, as judged by a study-validated question: “How would you characterize your ability to develop and maintain an erection adequate for satisfying sexual intercourse?” The answer possibilities were “never,” “sometimes,” “usually,” and “almost often or almost always.” We classified ED as individuals who replied “sometimes able” or “never able” for our main outcome. In the sensitivity analysis, only males who reported “never” being able to sustain an erection were considered to have ED (26). ED prevalence was conceived as an outcome variable.

The potential confounding factors that may affect the association between the TyG index and ED were identified in multivariate adjusted models. Covariates in our study included age (years), race, education level, poverty to income ratio (PIR), marital status (married or living with partner/single), alcohol consumption (drinking or not), cholesterol level (mg/dl), body mass index (BMI), smoking status (yes/no), hypertension, diabetes, asthma (27), coronary artery disease (26), testosterone (ng/ml) and estradiol (pg/ml).

Missing values:Numerous missing values for PIR, testosterone, BMI, and estrogen were transformed to categorical categories, and the missing values constituted their own group as dummy variables.

### Statistical methods

The NHANES study’s sampling weights, stratification, and clustering were applied to all statistical analyses to account for the complex, multi-stage sampling design used in selecting a representative non-institutionalized US population and to obtain accurate estimates of statistical significance that were not overstated. In accordance with NHANES analytic rules, new sample weight for the combined survey cycles was derived by dividing the 2-year weights for each cycle by 2 (28). Using the survey design R package in the R programming language and the weights supplied by the dataset, we interpreted the sophisticated multi-stage stratified sampling method of NHANES. We describe demographic and clinical parameters based on the presence or absence of ED in the examined population.

Categorical variables were reported as weighted survey means and 95 percent confidence intervals, while continuous variables were expressed as weighted survey means and 95 percent confidence intervals. To analyze differences between the two groups, survey-weighted linear regression (for continuous variables) and survey-weighted chi-square tests (for categorical variables) were used to continuous data. To exclude the problem of cointegration, we used the cointegration test, when VIF greater than 5 was considered to have cointegration problem, the covariate was removed. After screening for valid covariates based on guidelines (29). In model 1, no variables were modified. Model 2 was modified for age, race, marriage, and degree of education. The third model was modified for all variables. Using smoothed curve fitting (penalized spline approach) and generalized additive model regression (GAM) regression, the connection between TyG index and ED was evaluated further. The likelihood ratio test was used to find inflection point values when a nonlinear connection was found to exist. Next, stratified multiple regression analyses were conducted by age, race, BMI, hypertension, and diabetes. P < 0.05 was considered statistically significant. All analyses were performed using Empower software (www.empowerstats.com); and R version 4.0.2 (http://www.R-project.org, The R Foundation).

## Results

### Elevated TyG index levels in the ED group

The baseline demographic features of the enrolled individuals are detailed in **Table 1**, along with the weighted distribution of characteristics of the study population for the whole sample. TyG index was 8.6 (95% CI: 8.6, 8.7) in the non-ED population and 9.0 (95% CI:8.9,9.0) in the ED population. The ED population had a higher TyG index, p<0.001. In the ED group, age, hypertension, diabetes, and coronary heart disease were considerably greater than in the control group. In the ED group, the prevalence of age, hypertension, diabetes, and coronary heart disease was considerably greater than in the non-ED group, although the education level and testosterone level were significantly lower.

**Table 1:** Baseline characteristics of participants, weighted.

Characteristic	Non-ed formers	Ed formers	P-value
	N = 2560	N = 606	
Age(years)	40.4 (39.8,40.9)	53.9 (53.0,54.9)	<0.0001
Serum Cholesterol (mg/dl)	201.9 (200.0,203.9)	202.9 (198.0,207.8)	0.7294
Serum HDL-c (mg/dl)	47.1 (46.4,47.8)	45.8 (44.8,46.9)	0.0258
TyG index	8.6 (8.6,8.7)	9.0 (8.9,9.0)	<0.0001
Race(%)			0.2159
Mexican American	7.9 (6.0,10.2)	8.1 (5.1,12.7)	
Other Hispanic	4.1 (2.6,6.3)	6.5 (3.0,13.6)	
Non-Hispanic White	74.2 (70.0,77.9)	71.6 (64.0,78.1)	
Non-Hispanic Black	9.8 (7.8,12.2)	10.0 (7.3,13.6)	
Other Race	4.1 (3.1,5.4)	3.8 (2.5,5.8)	
Education Level(%)			<0.0001
Less than high school	13.1 (11.9,14.6)	27.1 (21.9,33.0)	
High school	27.8 (25.6,30.1)	23.1 (19.4,27.3)	
More than high school	59.1 (56.5,61.5)	49.8 (44.6,54.9)	
Marital Status(%)			<0.0001
Cohabitation	68.2 (65.5,70.9)	78.3 (74.6,81.6)	
Solitude	31.8 (29.1,34.5)	21.7 (18.4,25.4)	
Asthma(%)			0.0247
No	88.8 (87.3,90.1)	91.5 (89.4,93.2)	
Yes	11.2 (9.9,12.7)	8.5 (6.8,10.6)	
Alcohol(%)			0.0321
Yes	84.4 (80.1,88.0)	81.2 (76.5,85.2)	
No	15.6 (12.0,19.9)	18.8 (14.8,23.5)	
Diabetes(%)			<0.0001
Yes	3.8 (2.9,4.9)	22.6 (18.4,27.3)	
No	96.2 (95.1,97.1)	77.4 (72.7,81.6)	
High Blood Pressure(%)			<0.00001
Yes	20.5 (18.2,22.9)	47.8 (43.8,52.0)	
No	79.5 (77.1,81.8)	52.2 (48.0,56.2)	
Smoked			<0.0001
Yes	54.1 (51.1,57.0)	69.8 (65.7,73.6)	
No	45.9 (43.0,48.9)	30.2 (26.4,34.3)	
Coronary Artery Disease			<0.0001
Yes	2.2 (1.6,2.9)	11.6 (8.4,15.8)	
No	97.8 (97.1,98.4)	88.4 (84.2,91.6)	
PIR(%)			0.0064
<1.3	14.8 (12.9,16.9)	19.7 (15.3,25.0)	
≥1.3,<**3.5**	31.9 (29.4,34.4)	35.0 (30.3,39.9)	
≥3.5	48.3 (45.1,51.6)	41.1 (36.1,46.3)	
Unclear	5.0 (3.8,6.6)	4.2 (2.6,6.8)	
BMI(%)			<0.0001
<**25kg/m^2^**	30.1 (28.2,32.0)	21.0 (17.1,25.6)	
25-29.9kg/m^2^	40.7 (38.5,42.9)	38.2 (33.7,42.8)	
≥30kg/m^2^	28.4 (26.1,30.8)	38.9 (34.0,44.1)	
Unclear	0.9 (0.5,1.6)	1.8 (1.1,3.2)	
Testosterone (ng/ml)			0.0005
Tertile 1	4.8 (3.8,6.2)	9.8 (7.0,13.6)	
Tertile 2	5.8 (4.8,7.1)	4.4 (2.9,6.6)	
Tertile 3	5.4 (4.3,6.8)	3.8 (2.3,6.3)	
Unclear	83.9 (81.8,85.8)	82.0 (77.8,85.5)	
Estradiol (pg/ml)			0.6254
Tertile 1	5.2 (4.4,6.1)	6.6 (4.5,9.7)	
Tertile 2	5.2 (4.2,6.4)	6.2 (3.7,10.0)	
Tertile 3	5.7 (4.4,7.4)	5.2 (3.1,8.7)	
Unclear	83.9 (81.8,85.8)	82.0 (77.8,85.5)	

For continuous variables: survey-weighted mean (95% CI), P-value was by survey-weighted linear regression.

For categorical variables: survey-weighted percentage (95% CI), P-value was by survey-weighted Chi-square test.

### ED prevalence was associated with a higher TyG index

According to the collinearity check results, the VIF value for the all covariates were less than 3, and the data were not subject to collinearity problem. The findings of logistic regression demonstrated a significant relationship between TyG index and ED prevalence. In the fully adjusted model (model 3), each unit increase in TyG index was linked with a 25% greater risk of erectile dysfunction (OR=1.25, 95% CI:1.03, 1.52).In addition, we converted the TyG index from a continuous (continuous) variable to a categorical (categorical) variable for sensitivity analysis. As shown in **Table 2**, there was a trend towards increased asthma prevalence in the high quartile group compared with the TyG index in the low quartile group, but without an effect value.

**Table 2:** Analysis between TyG index with ED prevalence.

Characteristic	Model 1 OR (95%CI)	Model 2 OR (95%CI)	Model 3 OR (95%CI)
TyG index	1.70 (1.51, 1.92)	1.36 (1.18, 1.56)	1.25 (1.03, 1.52)
Lower	1	1	1
Higher	1.90 (1.58, 2.28)	1.34 (1.09, 1.65)	1.11 (0.87, 1.42)

Model 1, no covariates were adjusted.

Model 2, Model 1+age,race,education and marital status were adjusted.

Model 3, Model 2+,diabetes,blood pressure, PIR, smoked, alcohol use, serum cholesterol, HDL-c, asthma, coronary artery disease, testosterone and estradiol were adjusted.

### TyG’s dose-response and threshold effects on ED prevalence

**Figure 1:**
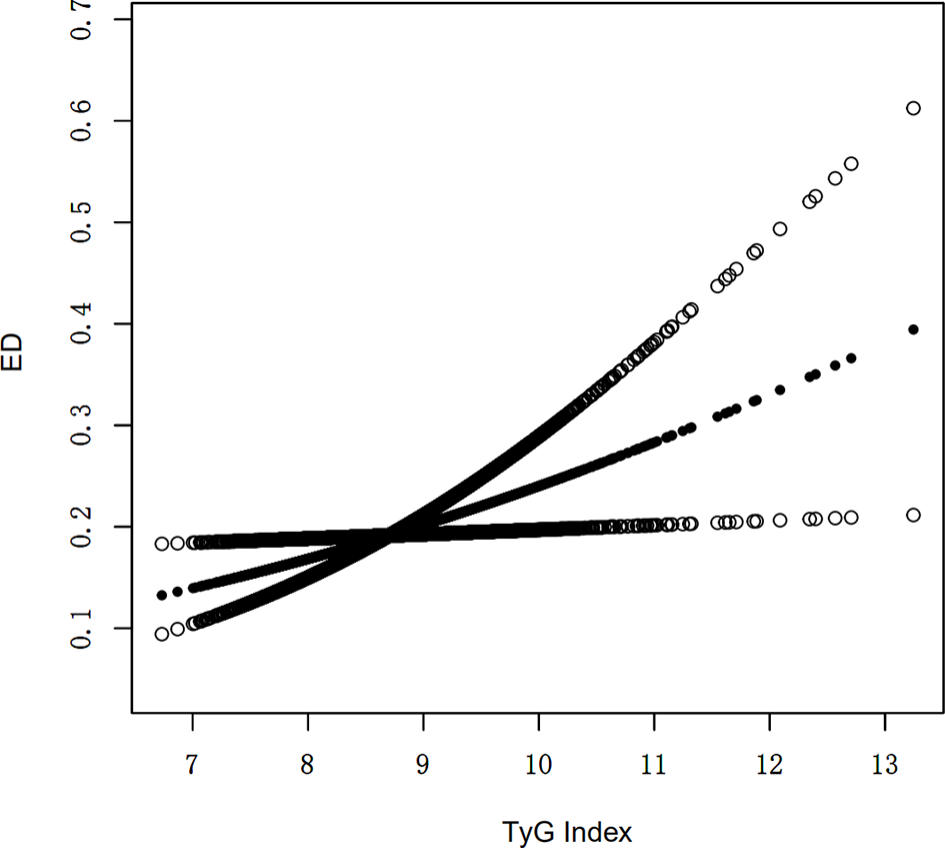
Density dose-response relationship between TyG index with ED prevalence. The area between the upper and lower dashed lines is represented as 95% CI. Each point shows the magnitude of the TyG index and is connected to form a continuous line. Adjusted for all covariates except effect modifier.

An additive generalized model and smoothed curve fitting were used to explore the relationship between *TyG* index and ED. According to our results, there is a positive linear correlation between *TyG* index and ED (**Figure 1**).

### Subgroup analysis

Subgroup analyses were conducted in order to examine the robustness of the association between METS-IR and ED. Based on the results of the subgroup analysis, age ≥50 years (OR=1.35, 95% CI:1.05, 1.74), the Mexican American (OR=1.50, 95% CI:1.00, 2.23), BMI 25-29.9 kg/m^2^ (OR=1.48, 95% CI:1.08, 2.01) and diabetes (OR=1.81, 95% CI:1.18, 2.78) categories were related with a higher prevalence of ED. In addition, we examined the relationships with age, race, BMI, hypertension, and type 2 diabetes. No statistically significant associations were detected with interactions (p > 0.05 for interactions) (**Table 3**).

**Table 3:** Analysis between TyG index with ED prevalence.

Characteristic	Model 1 OR (95%CI)	Model 2 OR (95%CI)	Model 3 OR (95%CI)	P for Interaction*
Age				0.91
Age<50	1.41 (1.14, 1.73)	1.30 (1.04, 1.62)	1.20 (0.87, 1.65)	
Age≥50	1.45 (1.22, 1.72)	1.50 (1.25, 1.79)	1.35 (1.05, 1.74)	
Race				0.08
Mexican American	1.57 (1.22, 2.02)	1.30 (0.99, 1.71)	1.50 (1.00, 2.23)	
Other Hispanic	1.52 (0.98, 2.37)	1.54 (0.96, 2.48)	1.54 (0.61, 3.91)	
Non-Hispanic White	1.87 (1.56, 2.24)	1.56 (1.27, 1.92)	1.36 (0.99, 1.86)	
Non-Hispanic Black	1.65 (1.25, 2.18)	1.03 (0.74, 1.45)	0.75 (0.47, 1.20)	
Other Race	1.64 (0.83, 3.22)	1.25 (0.58, 2.73)	2.09 (0.37, 11.82)	
BMI				0.30
<25kg/m^2^	1.84 (1.41, 2.40)	1.15 (0.84, 1.56)	0.89 (0.58, 1.38)	
25-29.9kg/m^2^	1.71 (1.40, 2.08)	1.42 (1.13, 1.78)	1.48 (1.08, 2.01)	
≥30kg/m^2^	1.53 (1.24, 1.88)	1.45 (1.14, 1.85)	1.35 (0.97, 1.90)	
Unclear	0.45 (0.14, 1.47)	0.23 (0.03, 1.58)	8394.21 (0.00, Inf)	
High Blood Pressure				0.80
Yes	1.41 (1.16, 1.70)	1.45 (1.16, 1.80)	1.32 (0.97, 1.79)	
No	1.65 (1.40, 1.94)	1.25 (1.03, 1.50)	1.24 (0.96, 1.60)	
Diabetes				0.10
Yes	1.05 (0.80, 1.39)	1.22 (0.89, 1.68)	1.81 (1.18, 2.78)	
No	1.45 (1.26, 1.68)	1.16 (0.98, 1.38)	1.15 (0.91, 1.45)	

Model 1, no covariates were adjusted.

Model 2, Model 1+age,race,education and marital status were adjusted.

Model 3, Model 2+,diabetes,blood pressure, PIR, smoked, alcohol use, serum cholesterol, HDL-c, asthma, coronary artery disease, testosterone and estradiol were adjusted. The subgroup analysis was stratified by race, age BMI, diabetes and HBP, not adjusted for the stratification variable itself.

*means only in model 3.

### Sensitivity analysis

**Figure 2:**
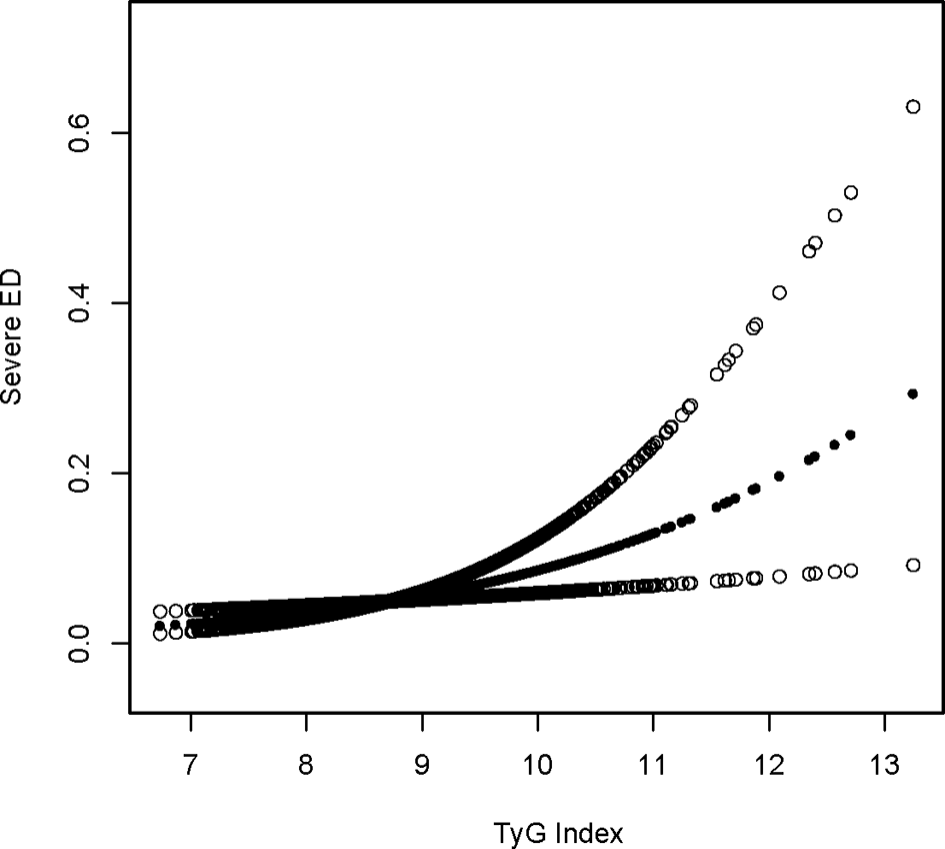
Sensitivity density dose-response relationship between TyG index with serious ED prevalence. The area between the upper and lower dashed lines is represented as 95% CI. Each point shows the magnitude of the TyG index and is connected to form a continuous line. Adjusted for all covariates except effect modifier.

Using a tighter definition of ED for those who “never” maintained an acceptable erection(serious ED), logistic regression revealed that the OR increased in model 3 after controlling for all factors when using a stricter definition of ED for those who “never” maintained an adequate erection. A one-unit increase in the TyG index was related to a 65 percent increase in ED prevalence in the higher group relative to the lower group (OR = 1.65, 95 percent CI: 1.07, 2.53). Lastly, the association between the TyG index and ED was reexamined using generalized additive modeling and smoothing fit, and the linearly positive relationship between the TyG index and stricter ED prevalence persisted (**Table 4**; **Figure 2**).

**Table 4:** Sensitivity analysis between TyG index with ED prevalence.

Characteristic	Model 1 OR (95%CI)	Model 2 OR (95%CI)	Model 3 OR (95%CI)
TyG index	1.79 (1.48, 2.17)	1.50 (1.20, 1.87)	1.58 (1.16, 2.16)
Lower	1	1	1
Higher	2.42 (1.70, 3.43)	1.73 (1.20, 2.49)	1.65 (1.07, 2.53)

Model 1, no covariates were adjusted.

Model 2, Model 1+age,race,education and marital status were adjusted.

Model 3, Model 2+,diabetes,blood pressure, PIR, smoked, alcohol use, serum cholesterol, HDL-c, asthma, coronary artery disease, testosterone and estradiol were adjusted.

### Sensitivity subgroup analysis

Subgroup analysis for the more stringent ED-defined population(serious ED) revealed that the OR became stronger in the age ≥50 years group (OR=1.80,95% CI:1.26, 2.58), the Mexican American group (OR=2.24,95% CI:1.22,4.11), and the diabetes group (OR=2.00,95% CI:1.13, 3.54), in addition Elevated TyG index was associated with increased ED prevalence in newly added subgroups: BMI ≥30 kg/m^2^ (OR=1.91,95% CI:1.11, 3.24); non-diabetic group (OR=1.54, 95% CI:1.02,2.33), non-hypertensive group (OR=1.77, 95% CI:1.13,2.77), non-Hispanic white group (OR=2.00, 95%CI:1.17,3.41) (**Table 5**).

**Table 5:** Sensitivity subgroup analysis between TyG index with ED prevalence.

Characteristic	Model 1 OR (95%CI)	Model 2 OR (95%CI)	Model 3 OR (95%CI)	P for Interaction*
Age				0.56
Age<50	1.38 (0.90, 2.11)	1.23 (0.76, 1.99)	1.09 (0.56, 2.14)	
Age≥50	1.63 (1.28, 2.08)	1.62 (1.26, 2.07)	1.80 (1.26, 2.58)	
Race				0.16
Mexican American	1.69 (1.15, 2.49)	1.54 (1.03, 2.31)	2.24 (1.22, 4.11)	
Other Hispanic	1.39 (0.66, 2.91)	1.90 (0.53, 6.72) 0	6.09 (0.28, 131.09)	
Non-Hispanic White	2.07 (1.56, 2.74)	1.95 (1.39, 2.75)	2.00 (1.17, 3.41)	
Non-Hispanic Black	1.40 (0.83, 2.35)	1.02 (0.56, 1.85)	0.78 (0.34, 1.78)	
Other Race	1.23 (0.19, 8.04)	0.76 (0.07, 8.42)	0.01 (0.00, Inf)	
High Blood Pressure				0.50
Yes	1.43 (1.09, 1.88)	1.48 (1.08, 2.02)	1.54 (0.98, 2.43)	
No	1.85 (1.39, 2.47)	1.53 (1.11, 2.12)	1.77 (1.13, 2.77)	
Diabetes				0.11
Yes	1.02 (0.72, 1.46)	1.28 (0.84, 1.97)	2.00 (1.13, 3.54)	
No	1.63 (1.26, 2.11) 0	1.36 (1.01, 1.83)	1.54 (1.02, 2.33)	
BMI				0.48
<25kg/m^2^	1.62 (1.01, 2.60)	1.02 (0.57, 1.82)	1.07 (0.49, 2.31)	
25-29.9kg/m^2^	1.67 (1.20, 2.31)	1.41 (0.97, 2.06)	1.61 (0.96, 2.71)	
≥30kg/m^2^	1.92 (1.39, 2.64)	1.82 (1.26, 2.63)	1.90 (1.11, 3.24)	
Unclear	1.58 (0.42, 5.97)	4.56 (0.34, 60.23)	0.00 (0.00, Inf)	

Model 1, no covariates were adjusted.

Model 2, Model 1+age,race,education and marital status were adjusted.

Model 3, Model 2+,diabetes,blood pressure, PIR, smoked, alcohol use, serum cholesterol, HDL-c, asthma, coronary artery disease, testosterone and estradiol were adjusted. The subgroup analysis was stratified by race, age BMI, diabetes and HBP, not adjusted for the stratification variable itself.

*means only in model 3.

## Discussion

In this nationally representative cross-sectional research examining the connection between TyG index and erectile dysfunction in males, we discovered that high levels of TyG index were related with a higher prevalence of erectile dysfunction among adult men in the United States. Following sensitivity analysis, the impact values strengthened. According to our knowledge, this is the first research using the NHANES database to examine the link between TyG index and erectile dysfunction in the general population.

Previous research has shown variable degrees of relationship between erectile dysfunction and numerous MetS components, including obesity, hypertension, hyperglycemia, dyslipidemia, body mass index, increased cholesterol, and HDL cholesterol (30, 31). The TyG index is a novel indicator derived from TG and fasting glucose that has lately been regarded as an intuitive and reliable predictor of IR to help in clinical decision making (32). TyG index is applicable to all patients, unlike HOMA-IR, since insulin is not included into the computation (33). Correlational studies imply that the primary pathophysiology of MetS is IR, and that diabetes is a significant MetS component (34). Stringent blood glucose management does minimize the prevalence of erectile dysfunction in individuals (35). The prevalence of erectile dysfunction increases proportionally with the duration of diabetes and glycemic management. In our research, greater TyG index in the diabetic group was related with an increased prevalence of ED in subgroup analysis, with considerably larger impact values than in the non-diabetic group (35). Moreover, in the current research, subgroup analysis revealed that a raised TyG index was linked with a higher prevalence of ED in the 50-year-old and Chicano populations, with greater impact values following sensitivity analysis. It is already common knowledge that aging increases the occurrence of ED. In the United States, the prevalence of ED diagnosis or treatment rose from 18-29 years (0.4%) to 60-69 years (11.5%) (36) and was related with an increase in the prevalence of comorbidities. In the current study, we confirmed by interaction test that age did not have a significant effect on the outcome of the association, and we believe that a reasonable explanation for this is that an increase in TyG index at age 50 years may respond to a greater degree of IR, an increased prevalence of comorbidities associated with IR, and ultimately an increase in the prevalence of ED *via* increased comorbidities. Additionally, the influence of race on ED has been documented. Hispanic males in the United States are at greater risk for erectile dysfunction, according to an NHANES study (37), another research from the Boston-based American Association for the Advancement of Science Another Boston, United States research (38) reported a greater frequency of ED among Hispanic males. The main finding of this study was an association between elevated TyG index and increased prevalence of ED in Mexican Americans, whereas some studies have included Mexican Americans in Hispanic studies (39), therefore our findings are consistent with those previously published. Finally, it is important to note that in the sensitivity analysis, the non-hypertensive group had higher TyG index impact values on ED. This intriguing phenomena may be difficult to grasp, yet investigations in other domains have shown comparable findings. A study from Iran found that IR in the non-hypertensive population led to cardiovascular disease, while IR in the hypertensive population had no such effect (40). Another Japanese study found that raising IR levels in non-diabetic adults increased the risk of coronary heart disease and stroke (41). This conclusion indicates that, despite the diverse research population, our results may be valid to some degree. This discovery, however, is constrained by the short sample size and requires confirmation by a prospective multicenter investigation with a high sample size.

To date, there are few reports on the effect of insulin resistance on the occurrence of ED. In a research demonstrated that ED patients with IR had lower IIEF-5 ratings and greater ED severity than ED patients without IR (42). Insulin resistance has been identified as a potential underlying cause of erectile dysfunction in young individuals (43). Insulin enhances NO generation *via* promoting the expression and activity of endothelial-type nitric oxide synthase (ENOS), according to *in vitro* and *in vivo* investigations (21, 22, 24). In contrast, insulin resistance is characterized by poor vascular NO synthesis and reduced insulin-induced vasodilation (21), as well as a reduction in basal NO production (11). In addition, the raised FFA levels associated with a high-fat diet decrease NO generation by downregulating the 5’-adenosine monophosphate-activated protein kinase (AMPK)-phosphatidylinositol 3-kinase (PI3K)-eNOS pathway in endothelial cells (44).The main characteristic of endothelial dysfunction is reduced nitric oxide (NO) production, which can lead to difficulties in smooth switching between vasodilation and constriction (44). In contrast, vascular endothelial dysfunction is one of the core mechanisms of ED development (17).Inflammation and oxidative stress also play a significant role in the relationship between IR and impotence. Whereas insulin resistance decreases NO release and synthesis and increases oxidative stress and inflammatory cytokines in endothelial cells, correlative experiments indicate that in insulin-resistant obese rats, IR leads to increased expression of vascular endothelin-b receptors, which play a role in increased reactive oxygen species production, endothelial dysfunction, and increased vasoconstriction in erectile tissue (18). All of the aforementioned studies indicate that IR plays a crucial role in the etiology of ED, which may explain the association between TyG index and erectile dysfunction in males.

Our research offers several benefits. The NHANES 2001-2004 is a representative sample of the US population that carefully adheres to a well-designed research procedure with rigorous quality assurance and quality control. In addition, a number of sensitivity studies that support our core analysis confirm our conclusions. Our research certainly has some limitations. We were unable to establish a causal association between the TyG index and erectile dysfunction in males since our analysis was based on the NHANES database, which is a cross-sectional study. Second, the diagnosis of erectile dysfunction in men was based on a questionnaire, which was unable to accurately determine the severity of erectile dysfunction and was subject to recall bias; third, detailed clinical variables such as individual medication history and type of erectile dysfunction were not disclosed in the database and require further investigation. Consequently, a multicenter RCT investigation is required to corroborate our findings further. This research confirms the association between the TyG index and erectile dysfunction despite these limitations.

## Data availability statement

The original contributions presented in the study are included in the article/Supplementary Material. Further inquiries can be directed to the corresponding authors.

## Ethics statement

The studies involving human participants were reviewed and approved by The NCHS Research Ethics Review Committee approved the NHANES survey protocol (https://www.cdc.gov/nchs/nhanes/irba98.htm), and all participants of the study provided informed written consent. The NHANES database is open to the public and therefore the ethical review of this study was exempt. The patients/participants provided their written informed consent to participate in this study.

## Author contributions

Data analysis and manuscript writing: LL, HY, and MC; Study design and statistical advice: LL, HY, and WDa; Manuscript editing: HY, WDa, YC, JW, and MC; Validation and review: HL, WDi, YL, and JW; Quality control: LT, JW, and MC. All authors agreed on the journal to which the article was to be submitted and agreed to take responsibility for all aspects of the work. All authors contributed to the article and approved the submitted version.

## Funding

This work was supported by the Natural Science Foundation of Anhui Province (2108085MH269).

## Acknowledgments

We would like to thank all NHANES participants and staff. We are also grateful to Dr Xudong Shen for providing design ideas and statistical methodology advice.

## Conflict of interest

The authors declare that the research was conducted in the absence of any commercial or financial relationships that could be construed as a potential conflict of interest.

## Publisher’s note

All claims expressed in this article are solely those of the authors and do not necessarily represent those of their affiliated organizations, or those of the publisher, the editors and the reviewers. Any product that may be evaluated in this article, or claim that may be made by its manufacturer, is not guaranteed or endorsed by the publisher.

## Abbreviations

NHANES, National Health and Nutrition Examination Survey; BMI, body mass index; PIR, ratio of family income to poverty; NCHS, National Center for Health Statistics; CI, confidence interval; OR, odds ratio; MetS, metabolic syndrome; IR, insulin resistance; TG, Triglyceride; TC, Cholesterol; FPG, fasting plasma glucose; TyG index, triglyceride glucose index; ED, erectile dysfunction; HBP, Hypertension.
